# Infographics on signs and symptoms of metastatic (secondary) breast cancer can empower women with a breast cancer diagnosis

**DOI:** 10.3389/fpsyg.2024.1403114

**Published:** 2024-07-12

**Authors:** Nazanin Derakshan, Joanne Taylor, Bethany Chapman

**Affiliations:** ^1^School of Psychology and Clinical Language Sciences, University of Reading, Reading, United Kingdom; ^2^METUP UK, Manchester, United Kingdom

**Keywords:** breast cancer, metastatic breast cancer, infographics, coping, anxiety

## Abstract

We investigated the usefulness of a metastatic (secondary) breast cancer Infographics designed to enhance knowledge about symptoms of metastatic breast cancer in women diagnosed with breast cancer. Women with a primary or metastatic diagnosis of breast cancer who had not been in receipt of the Infographics previously, were sent the Infographics and asked to complete a questionnaire measuring their views of the usefulness of the Infographics in a number of domains. They were also asked to complete questionnaires on, anxiety and depression, coping, emotion regulation strategies and perceived cognitive functioning. Results showed that women advocated the use of the Infographics in medical and health care settings, as well as its ability in equipping themwith the relevant knowledge on signs of recurrence, its benefits in empowering control and reducing fears and uncertainties regarding metastatic breast cancer. Exploratory analysis showed that individual differences in trait vulnerability to anxiety and in emotion regulation strategies modulated women’s responses suggesting the use of tailored approaches in the communication of the Infographics with patients. Our results point to the overall benefits of the Infographics in a number of domains. Implications for applications in healthcare settings are discussed.

## 1 Introduction

Breast cancer is the biggest malignancy in women worldwide with currently 7.8 million women living with a diagnosis of breast cancer (World Health Organization, n.d.). While medical advances have led to higher survival rates, the psychological cost of diagnosis and treatment side effects continue to adversely affect the overall well-being of women. In addition to physical suffering and pain, there are numerous psychological vulnerabilities which can impair quality of life (Carreira et al., 2018). Cancer related cognitive decline (Ahles and Root, 2018), vulnerability to anxiety and depression (Wang et al., 2020), impaired workability (Chapman et al., 2023) and fears of cancer recurrence (Butow et al., 2019) can be chronic comorbidities which can continue to pose significant challenges for many years in survivorship (see Joly et al., 2019, for a review). Psychological interventions including group therapy and exercise have proven beneficial for promoting self-awareness of needs as well as better physical and psychological outcomes (Sebri et al., 2022). Unfortunately however, psychological support is not a standard provision in the cancer care pathway, and women can find it difficult and challenging to rebuild their lives or navigate their way moving forward.

In the UK, every 10 min a woman is diagnosed with breast cancer (Breast Cancer Now, 2023a,b). Early screening Survival rates for primary breast cancer have increased due to early screening and treatments. However, breast cancer is still the leading cause of death in women under the age of 50 (Breast Cancer Now, 2023a,b). Recent figures predict that 1 in 3 women diagnosed with primary breast cancer will go on to receive a metastatic diagnosis (also known as stage IV breast cancer or secondary breast cancer) (Breast Cancer Org, 2024) which is incurable. Metastatic breast cancer is diagnosed when the cancer cells have metastasized (spread) outside of the breast(s) into single or multiple sites around the body including but not limited to the bones, brain, liver, or lungs (Berman et al., 2013).

Worryingly, reports (Breast Cancer Now, 2020) have suggested that 24% of women treated for primary breast cancer in the UK consult their General Practitioner about symptoms of metastatic breast cancer at least three times before receiving their diagnosis and 41% who’d discussed their symptoms with a healthcare professional felt they were not taken seriously. In fact, 20% of women were treated for a different condition prior to their diagnosis of metastatic breast cancer. These findings suggest that healthcare professionals are not fully equipped with the relevant knowledge of metastatic breast cancer symptoms and delays in diagnosis can increase disease progression. Relatedly, a substantial proportion of women in the UK are not briefed by their medical teams about the possible symptoms of metastatic breast cancer upon finishing their active treatment (surgery/chemotherapy and/or radiotherapy) for primary breast cancer.

Fear of cancer recurrence and/or progression to metastatic breast cancer is highly prevalent and can exist up to 10 years amongst survivors of breast cancer leading to clinical anxiety and depression (Ban et al., 2021; Tran et al., 2022). These fears are the most common unmet need of individuals living with a diagnosis of breast cancer (Simard et al., 2013; Butow et al., 2019). A large meta-analysis has confirmed that depression and anxiety can increase risk of breast cancer specific and all-cause mortality by up to 30% (Wang et al., 2020). Emotion regulation strategies such as expressive suppression or avoidance of threat can increase risk of depression through effects on the endocrine and immune systems (Guimond et al., 2019). Equally, hypervigilance for symptoms of breast cancer recurrence and metastasis can lead to misinterpretation of symptoms, unnecessary scans and appointments, and increase worry and anxiety (Tauber et al., 2019). Therefore, understanding individual differences in response to information on signs and symptoms of metastasis can help develop personalized programmes to meet individual needs when educating patients on the signs of recurrence and metastasis.

Educational tools that can increase knowledge and provide individuals with a sense of autonomy and control in managing cancer related symptoms are of paramount importance. In recent research, the use of Infographics as a means of communication with patients and medical practitioners has gained much interest in multiple health domains (Mc Sween-Cadieux et al., 2021). Infographics have the potential to disseminate knowledge more efficiently to a wide range of populations. There is evidence that Infographics have been effective in summarizing medical literature (Martin et al., 2019) as well as being a preferred tool in communicating information to patients and caregivers (Crick and Hartling, 2015). They can better capture attention and increase the efficiency by which cancer patients process information, locate and communicate their symptoms and concerns (Piil et al., 2023). Health Infographics have the potential to increase engagement and attention to bodily changes thus increasing self-focused attention which in turn has shown to promote loss aversion (Sebri et al., 2023). Individuals with greater self-focused attention made better decisions in uncertain situations due to being more responsive to internal bodily changes (Sebri et al., 2023). This finding points to the potential role of Infographics in improving self-awareness of bodily changes and has the potential to influence decision making and promote behaviour change.

**Figure 1:**
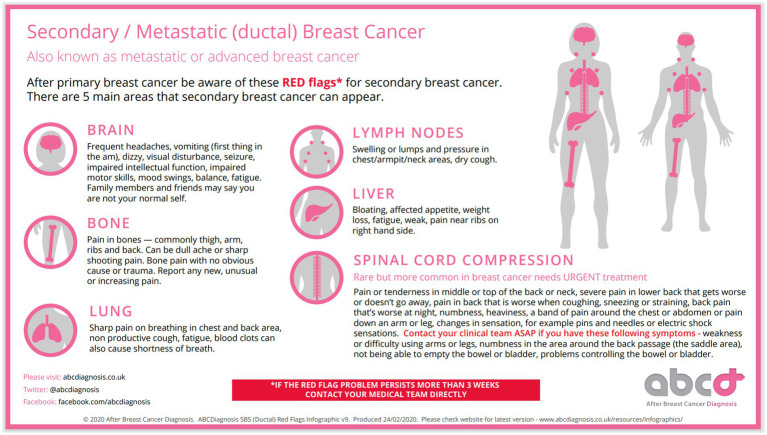
ABCDiagnosis SBS (Ductal) red flags infographics v9. Reproduced with permission from abcdiagnosis.

**Figure 2:**
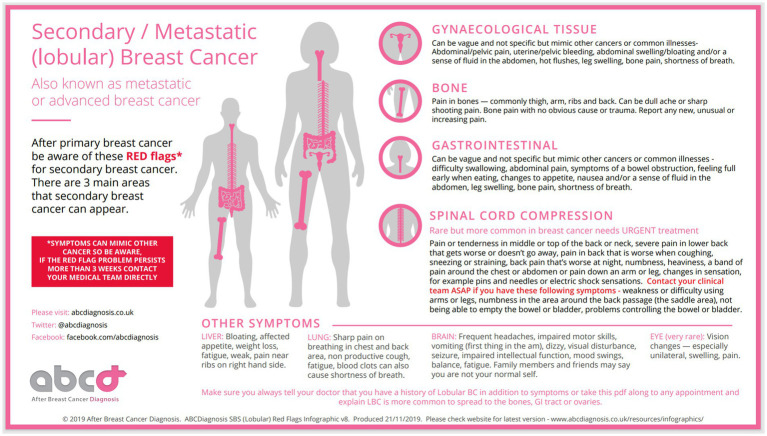
ABCDiagnosis SBS (Lobular) red flags infographics v8. Reproduced with permission from abcdiagnosis.

In the current study, which is the first of its kind, we investigated the perceived usefulness and benefits of a metastatic (secondary) breast cancer Infographics^1^ (see Figure 1 and Figure 2), developed by abcdiagnosis charity, the largest patient advocate charity for metastatic breast cancer in the UK. Our decision to use this Infographics was motivated by its detailed, inclusive and comprehensive pictorial and narrative approach to locations, signs and explanations of metastatic breast cancer breast, and its potential to be disseminated widely.

Women with either a primary or metastatic (secondary) diagnosis of breast cancer completed a questionnaire developed by the authors. This questionnaire included numerous items on the usefulness of the Infographics for patients and medical staff and its potentially empowering effect on patients’ sense of control over health concerns and management of fears of recurrence. A factor analysis was conducted to elucidate the pattern of responses to the items on the questionnaire. We also investigated the possible influence of individual differences in trait vulnerability to anxiety and emotion regulation strategies such as expressive suppression on the perceived utility of the Infographics. Understanding individual differences in response to education about signs of metastasis which can be potentially threatening can help tailor programmes for women who may be emotionally vulnerable and thus in greater need of psychological support.

## 2 Method

### 2.1 Participants

Women with a diagnosis of primary (*N* = 51) or metastatic (*N* = 59) breast cancer in the UK were recruited using volunteer response sampling via social media platforms Facebook, Twitter, and Instagram, including ‘Building Resilience in Breast Cancer Centre’ (BRiC; https://briccentre.co.uk/) and “METUPUK.”^2^

Inclusion criteria for primary breast cancer were: (1) aged 18 or over, (2) diagnosis of primary breast cancer, (3) post-active treatment for chemotherapy and/or radiotherapy, (4) can be receiving medication for hormone therapy (i.e., Tamoxifen) or target therapy (i.e., Herceptin) or psychopathology. Inclusion criteria for metastatic breast cancer: (1) age 18 or over, (2) diagnosis of metastatic breast cancer, (3) can be receiving radiotherapy and/or chemotherapy, hormone therapy or target therapy and medication for psychopathology. Exclusion criteria was age under 18.

Reasons for not participating in the study after registering an interest included receiving active treatment for primary breast cancer (*N* = 6) and being newly diagnosed with primary breast cancer (*N* = 1).

## 3 Materials

### 3.1 Demographic questionnaire

The demographics questionnaire (developed by the authors) comprised of 22 itemsand assessed women’s sociodemographic information, breast cancer history, diagnosis status of anxiety and/or depression and work-related characteristics. Information was self-reported.

### 3.2 Functional assessment of cancer therapy – cognitive scale - perceived cognitive impairment subscale (FACT-Cog-PCI, version 3)

The FACT-Cog-PCI is a reliable and valid subscale from the FACT questionnaire (Wagner et al., 2009), with 20 items measured on a 5-point Likert scale. PCI scores range from 0 to 80, with higher scores indicating less perceived cognitive impairment. Cronbach’s alpha for the current study was α = 0.97.

### 3.3 Depression, anxiety, and stress scale – 21 items (DASS-21)

The DASS-21 (Osman et al., 2012) is a reliable and valid questionnaire with 21 items measuring depression (7 items, range = 0–42), anxiety (7 items, range = 0–42) and stress (7 items, range = 0–42) experienced in the last week. Items are scored on a 4-point Likert scale from 0 to 3, with a total score ranging from 0 to 120 after multiplying the raw score by a factor of two. Higher scores represent a greater severity. Cronbach’s α were 0.92, 0.85, 0.91, 0.95 for depression, anxiety, stress and total, for the current study, respectively.

### 3.4 Rumination response scale - short form (RRS-SF)

The RRS-SF is a reliable and valid 10 item self-report questionnaire (Treynor et al., 2003). Items are rated on a 4-point Likert scale from 1 to 4, with the total score ranging from 10 to 40. Higher scores represent higher rumination. Cronbach’s α for the current study was α = 0.87.

### 3.5 Brief COPE

The Brief COPE (Carver, 1997) comprising 28 items measures coping strategies (or behaviours). It is composed of three subscales: problem-focused coping, emotion-focused coping, and avoidant coping measured on a 4-point Likert scale from 1 to 4. Total scores for each are calculated by summing the responses and then dividing the score by the number of items answered. Cronbach’s α = 0.86, 0.75, 0.59, 0.88 for problem-focused, emotion-focused coping, avoidant coping, and total score, in this study, respectively.

### 3.6 Emotion regulation questionnaire

The ERQ is a reliable and valid questionnaire containing 10 items assessing an individual’s tendency to regulate their emotions (Gross and John, 2003). It is composed of two subscales: cognitive reappraisal and expressive suppression measured on a 7-point Likert scale ranging from 1 to 7. Total scores for each are calculated by summing the responses and dividing the number of items in that subscale. Higher scores reflect a greater frequency of using that emotion regulation strategy. Cronbach’s α = 0.85 and 0.82 for cognitive reappraisal and expressive suppression, in this study, respectively.

### 3.7 Impact of events scale – revised (IES-R)

The IES-R (Weiss and Marmar, 1997) comprising 22 items measures the level of post-traumatic stress disorder (PTSD) experienced in the last 7 days. It is composed of three subscales known as intrusions (8 items; scoring range = 0 to 32), hyperarousal (6 items; scoring range = 0 to 24), and avoidance (8 items; scoring range = 0 to 32) measured on a 5-point Likert scale from 0 to 4. Total score ranges from 0 to 88. Higher scores indicate greater PTSD symptoms. In the current study, Cronbach’s α = 0.91, 0.86, 0.87, and 0.94 for intrusions, hyperarousal, avoidance, and total scores, respectively.

### 3.8 Metastatic breast cancer infographics questionnaire

22 items were developed by the authors measuring relevant cognitive and emotional factors in metastatic breast cancer. These broadly ranged from attitudes towards knowledge of symptoms and effects on the control, self-management and empowerment over one’s health, the usefulness of the Infographics in seeking medical advice and early detection, its application in healthcare settings such as hospitals, clinics and for health-care professionals, and its impact on fears and uncertainties surrounding recurrence and progression of disease. The items were reviewed by a team of patient advocates from both METUPUK and The BRiC Centre. Seventeen items were measured on a 5-point scale from 0 to 4, with higher scores representing more positive responses. Two multiple-choice (i.e., yes, no, other) items were used to assess firstly whether women wanted to know about the signs and symptoms of metastatic breast cancer, recurrence or progression (should they wish to terminate the study if the answer was no) and secondly, whether women were satisfied with the level of information they were provided by their oncologist at the end of their primary breast cancer treatment. In addition, three short answer questions were given to gain feedback on the design content of the Infographics.

### 3.9 Procedure

Women who emailed in response to one of the advertisements were sent the study information, participant inclusion criteria, and a secure URL link to access the questionnaires and the Infographics programmed on the Gorilla Experimenter platform.^3^ Women consented online before completing the demographics questions followed by the Infographics questions and the other questionnaires. Women completed all questionnaires in a single session to ensure consistency in responses although were told they could take short comfort breaks. Upon completion, women received a £10 Amazon e-gift voucher.

## 4 Statistical analysis and results

Statistical analysis was performed using SPSS version 29. Outliers were assessed using histograms and box plots. Using Shapiro–Wilk normality was assessed. Exploratory factor analysis with principal component extraction and Oblimin factor rotation was performed on the data and resulted in three factors. These factors were used in the analysis. Simple frequencies (response percentages) were calculated for the three factors. Bootstrapped Pearson’s correlation analysis was performed between the questionnaires measuring individual differences and the three metastatic breast cancer Infographics factors.

### 4.1 Sample characteristics

Table 1 displays the demographic, breast cancer, and work-related characteristics of the 110 women recruited. Table 2 shows the descriptive summaries for the individual differences questionnaires completed by the participants.

**Table 1:** Participants sociodemographic, clinical and work-related characteristics.

	Primary breast cancer (*n* = 51)	Metastatic breast cancer (*n* = 59)
*Sociodemographic*		
Age	Mean = 52.6 (Range 35.0–67.0)	Mean = 53.2 (Range 37.0–72.0)
Education		
Secondary education	2 (3.9)	4 (6.8)
Further education	10 (19.6)	11 (18.6)
Higher education	39 (76.5)	41 (69.5)
Other		3 (5.1)
Ethnicity^a^		
White	44 (86.3)	57 (96.6)
Asian/Asian British	4 (7.8)	0 (0.0)
Black/African/Caribbean/Black British		1 (1.7)
Mixed/Multiple ethnic	1 (2.0)	
Other	1 (2.0)	
*Work*		
Employment type		
Paid work	39 (76.5)	26 (44.1)
Unpaid work	4 (7.8)	6 (10.2)
Not currently working	6 (11.8)	21 (35.6)
Other	2 (3.9)	6 (10.2)
*Clinical -Breast Cancer History*		
Age at primary diagnosis	Mean = 48.0 (Range 30–65)	Mean = 43.6 (Range 31–62)
Age at secondary diagnosis		Mean = 49.7 (Range 31–69)
Stage of breast cancer		
1	12 (23.5)	
2	20 (39.2)	
3	12 (23.5)	
4		59 (100.0)
I do not know	7 (13.7)	
Active treatment		
Yes^b^	5 (9.8)	46 (78.0)
No	45 (88.2)	13 (22.0)
Currently taking endocrine therapy		
Yes	33 (64.7)	34 (57.6)
No	18 (35.3)	25 (42.4)
Receiving/received Herceptin		
Yes	4 (7.8)	12 (20.3)
No	47 (92.2)	47 (79.7)
Diagnosis of anxiety and/or depression PRIOR to diagnosis of breast cancer	18 (35.3)	10 (16.9)
Condition		
Anxiety	5 (27.8)	2 (20.0)
Depression	5 (27.8)	4 (40.0)
Anxiety and depression	7 (38.9)	3 (30.0)
Other (anxiety-related condition)		1 (10.0)
Not stated	1 (5.6)	
Current diagnosis of anxiety and/or depression	16 (31.4)	14 (23.7)
Condition		
Anxiety	6 (37.5)	1 (7.1)
Depression	3 (18.8)	4 (28.6)
Anxiety and depression	5 (31.3)	6 (42.9)
Other (anxiety-related condition)	1 (6.3)	1 (7.1)
Not stated	1 (6.3)	2 (14.3)

Percentages are shown in parentheses.

a One participant in the primary group and one participant in the secondary group did not disclose their ethnicity.

b Whilst all women reported being post-active treatment for chemotherapy and/or radiotherapy at the time of recruitment, five women later stated that they were receiving these treatments while completing the study. The analysis included these women as excluding them did not affect the findings.

**Table 2:** Descriptive summaries for each of the self-report individual differences questionnaires.

	Primary breast cancer		Metastatic breast cancer	
	Mean (SD)	Range (minimum - maximum)	Mean (SD)	Range (minimum - maximum)
*Cognitive functioning*				
Perceived cognitive impairment (FACT-PCI)	37.9 (20.4)	(3.0–77.0)	42.2 (20.6)	(3.0–80.0)
Rumination (RRS-SF)	21.3 (6.6)	(10.0–35.0)	18.6 (5.8)	(10.0–33.0)
*Emotional well-being*				
Anxiety (DASS-21)	9.6 (9.6)	(0.0–42.0)	6.8 (8.1)	(0.0–38.0)
Depression (DASS-21)	12.2 (10.4)	(0.0–42.0)	11.7 (10.4)	(0.0–42.0)
Stress (DASS-21)	14.9 (11.4)	(0.0–42.0)	13.2 (9.8)	(0.0–42.0)
PTSD symptoms (IES-R)	26.6 (18.0)	(0.0–88.0)	24.4 (16.9)	(1.0–73.0)
*Emotional regulation* ^a^				
Cognitive reappraisal (ERQ)	4.8 (1.1)	(2.3–6.8)	5.0 (1.1)	(1.0–7.0)
Expressive suppression (ERQ)	3.6 (1.4)	(1.0–6.3)	3.5 (1.5)	(1.0–6.5)
*Coping strategies* ^a^				
Problem-focused coping (Brief COPE)	2.4 (0.6)	(1.0–3.8)	2.3 (0.7)	(1.0–3.9)
Emotion-focused coping (Brief COPE)	2.0 (0.5)	(1.0–3.2)	2.1 (0.5)	(1.3–3.3)
Avoidant coping (Brief COPE)	1.6 (0.3)	(1.0–2.8)	1.6 (0.4)	(1.0–2.4)
Coping Response Total (Brief COPE)	2.0 (0.4)	(1.0–2.86)	2.0 (0.4)	(1.1–2.8)

Standard deviations are shown in parentheses.

a Scores are based on the average (sum of items divided by the number of items answered).

### 4.2 Prior knowledge of metastatic symptoms

Of women with primary breast cancer, 60% (and 57% of women with metastatic breast cancer) reported that they had no prior knowledge of metastatic breast cancer symptoms and did not have the opportunity to discuss these with their oncologist or medical teams upon end of active treatment. Around 29% of women in each group reported they had little knowledge, leaving under 10% reporting having sufficient knowledge.

### 4.3 Women’s responses to the metastatic breast cancer infographics

Of the 110 women (primary and metastatic) recruited, 89.1% (total 98; primary 92.2% (47); metastatic 86.4% (51)) reported that they wanted to know about the signs and symptoms of metastatic breast cancer, recurrence or progression. The remaining analyses were carried out on this subset of women.

The factor analysis revealed three factors which were labelled according to their item loadings on the pattern matrix (see Table 3). The first factor loaded on items measuring ‘empowerment, taking control of one’s health, lessening worries and fears, coping better with uncertainties surrounding metastasis’ and was labelled as ‘***Feeling Empowered***’ (all factor loadings >0.66). The second factor loaded on items such as, ‘the need for the Infographics to be discussed with patients by oncologists and medical teams as well as General Practitioners’ and was labelled as ‘***Application to Medical and Healthcare Settings*’** (all factor loadings >0.8), and the third factor loaded on items such as ‘increasing knowledge, feeling more equipped, and seeking medical attention when necessary’, and was labelled as ‘***Feeling Equipped and Knowledgeable***’ (all factor loadings >0.5).

**Table 3:** Factor loadings obtained from the factor analysis.

Item	Factor		
	1	2	3
In your opinion, do you think knowing these signs will make you feel more equipped (prepared) to make decisions about your health?			**0.772**
In your opinion, do you think discussing this Infographic with your oncologist would make you feel knowledgeable about secondary breast cancer?			**0.846**
In your opinion, do you think that the information presented on the Infographic will make you feel more empowered?	**0.670**		0.306
Do you think this infographic will make you more in control of your health?	**0.721**		0.303
Do you think receiving this Infographic will make you worry less about secondary breast cancer or cancer progression?	**0.939**		
Do you think receiving this Infographic will make you feel less fearful about the possibility of secondary breast cancer or cancer progression?	**0.916**		−0.315
Do you think this Infographic would help you cope with the uncertainty surrounding the risk of secondary breast cancer or cancer progression?	**0.818**		
Do you think this Infographic will influence how often you think about the possibility of cancer recurrence, secondary breast cancer or cancer progression?	0.320		0.325
In your opinion, do you think receiving a copy of this Infographic will influence how likely you are to seek medical attention?			**0.539**
In your opinion, do you think this Infographic should be discussed by your oncologist so they can allude you to the different signs and symptoms when they provide you with a copy of this information?		**0.979**	
Would you have liked to receive a copy of this Infographic from your oncology team as part of your treatment summary?		**0.979**	
In your opinion do you think this Infographic should be given to GPs to help promote their awareness, understanding, knowledge, and learning of the signs and symptoms of secondary breast cancer?		**0.784**	

Bold figures indicate relevant loadings for the factor.

**Figure 3:**
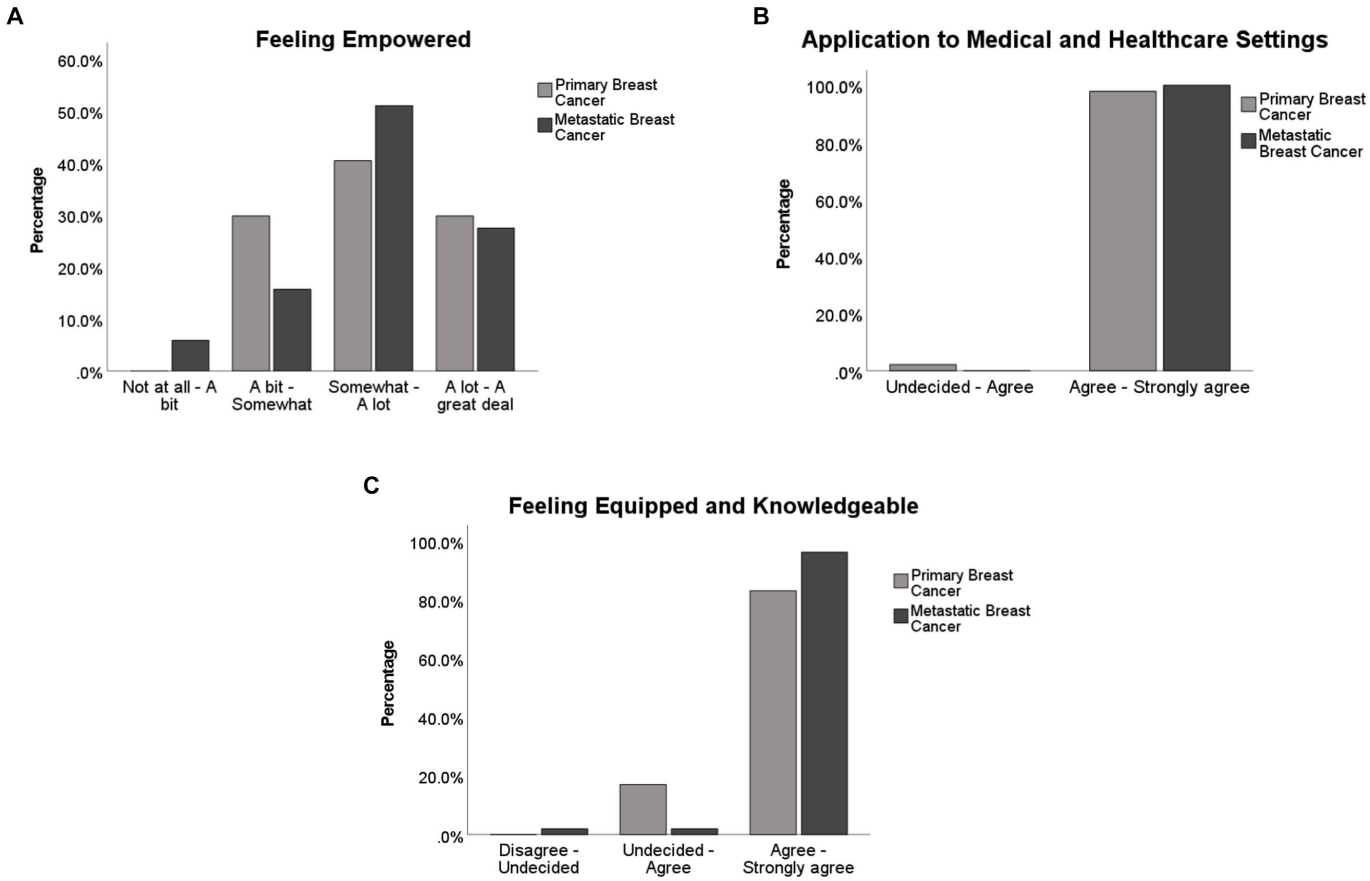
Percentage of participants responses on each of the three factors obtained from the factor analysis.

As shown in Figure 3A for the factor ‘Feeling Empowered’ the majority of women (70% primary and 79% metastatic) responded positively to the empowering effect of the Infographics on feeling more control, reducing worries, uncertainties and fears around metastases. Similarly Figure 3B shows that just under 100% of women with primary and 100% of women with metastatic breast cancer endorsed the application of the Infographics in health care settings and the opportunity to discuss the Infographics with medical practitioners. In the same fashion, women believed that the Infographics would make them knowledgeable, more equipped and likely to seek medical attention when necessary (Figure 3C).

### 4.4 Correlations between individual differences and infographics factors

Correlations were found between ‘feeling empowereds’ and the Anxiety subscale of the DASS scale, *r* = 0.28, *N* = 98, *p* = 0.005. Similar figures were obtained when this relationship was investigated for each of the primary and metastatic groups independently (*ps* < 0.05). Individuals with higher levels of anxiety perceived the positive impact of the Infographics on reducing their fears and worries about metastatic breast cancer symptoms with greater benefit. The only other relationship significant was between ‘feeling empowered’ and the Expression Suppression subscale of the Emotion Regulation Questionnaire only for the metastatic breast cancer group, *r* = −0.3, *N* = 51*, p* < 0.05 indicating that individuals with suppressive emotion regulation strategies are less likely to view the Infographics as empowering No other correlation coefficient was significant (all *ps* > 0.1).

## 5 Discussion

Women with a diagnosis of breast cancer are at an increased risk of anxiety and depression which can increase risk of mortality (see Wang et al., 2020, for a review). Fear of recurrence or progression of disease can be fueled by uncertainties and lack of knowledge of metastatic breast cancer symptoms. In the UK, upon ending treatment for primary breast cancer, women are usually not briefed on symptoms of metastatic breast cancer mainly due to the assumption that it can increase fears of recurrence at a time when women are keen to put the cancer behind them and move forward. However around a third of women with early stage breast cancer can develop secondary breast cancer which is incurable (Breast Cancer Org. nd). Fear of recurrence continues to be an unmet need and can linger for many years active treatment for primary breast cancer (Tran et al., 2022).

To our knowledge, our study is the first to investigate the perceived benefits and usefulness of a metastatic breast cancer Infographics aiming to enhance knowledge of metastatic breast cancer symptoms for women diagnosed with breast cancer. Our results show that a significant majority wanted to be educated about symptoms of metastatic breast cancer. Our participants largely agreed the Infographics would help them feel empowered and more in control of their health. Perceived lack of control in individuals with a history of cancer is common and linked with emotional vulnerability and fears surrounding recurrence and metastasis (Brandão et al., 2017). Our results suggest that knowledge of metastatic breast cancer symptoms can foster a sense of perceived control over health and well-being. Perceived control is negatively related to anxiety and depression in women with a history of breast cancer (Brandão et al., 2017) indicating its positive influence on quality of life. Our findings imply the Infographics can potentially help manage fears and anxieties surrounding recurrence and metastasis with implications for increased perceptions of control.

Research has shown survivors of breast cancer are highly motivated to participate and be included in psycho-educational programmes and interventions because of a main desire to improve their physical and psychological health outcomes (Sebri et al., 2022) as well as contributing to research. In addition, there is evidence that survivors of breast cancer are highly aware of their psychological needs (Sebri et al., 2022). Understanding the needs of survivors of breast cancer in terms of the educational tools they require to manage their fears and uncertainties surrounding metastatic breast cancer symptoms can help professionals devise more effective strategies for care and tailored support. In the current study, women strongly agreed that the Infographics should be included in medical and health care settings strongly advocating the opportunity to discuss symptoms of metastatic breast cancer with their medical practitioners and oncologist. This finding shows that women agree the Infographics will equip them with the knowledge they need to seek medical attention when necessary. The ability to make informed decisions increases confidence and self-esteem, which are naturally reduced after a cancer diagnosis. Our results indicate patients’ desire to be included in discussions surrounding metastatic breast cancer symptoms which can improve aid in decision making processes. Relatedly, these findings emphasise the need for medical practitioners to equip themselves with relevant knowledge of recurrence and metastasis sufficiently to avoid unnecessary delays in diagnosis as is currently the case (Breast Cancer Now, 2020). We suggest that the Infographics is discussed with patients alongside the end of treatment summary which is provided at the end of active treatment for primary breast cancer.Whilst our findings largely favoured the usefulness of the Infographics in a number of practical and psychological domains, individual differences modulated some of these views. The negative relationship between expressive suppression and perceived impact of the Infographics on feeling empowereds was found in the metastatic breast cancer group. Research has highlighted that expressive suppression is usually considered a maladaptive coping mechanism with harmful consequences, increasing risk of depression and avoiding painful situations which can encourage delays in seeking medical attention increasing risk of untreatable outcomes (Teng et al., 2022). This implies that for women who adopt an avoidant coping style, special care and attention with appropriate psychological support is required to help them accept and manage their fears and seek medical input.

In both women with primary and metastatic breast cancer higher anxiety scores were positively related to the empowering impact of the Infographics on reducing fears, worries and uncertainties regarding metastatic breast cancer. This finding may seem counter intuitive at first glance as anxiety has often been associated with avoidance of threat (i.e., avoiding signs of recurrence). However, intolerance of uncertainty features largely in high anxious individuals leading to catastrophising and clinical levels of worry and depression (Carleton et al., 2012). Our findings suggest that the Infographics can help manage the uncertainties surrounding metastatic breast cancer by providing the knowledge and power to increase certainty around decision making more effectively. However, we suggest this result is taken with caution and that women with a greater susceptibility to anxiety and depression have extra support in place when discussing symptoms of metastatic breast cancer with their medical teams.

### 5.1 Limitations

Our sample may be limited in ethnic representation as it was predominantly white and women from other ethnic backgrounds were underrepresented. Approximately one quarter of African women and black Caribbean women are diagnosed with advanced breast cancer (stage 3 or 4) (Breast Cancer Now, 2024) and as such are a highly relevant population to be considered in future research on education of signs of metastatic breast cancer.

Our participant sample was also well-educated. Our study was cross-sectional meaning that it only provides a single snapshot of women’s perceived views of these Infographics and their effectiveness at the time of completing the study. We recommend future research includes a randomised control trial comparing women who receive these Infographics as part of the end-of-treatment summary to a control group (e.g., women who have not received these Infographics).

## 6 Conclusion

Educating women about the symptoms of metastatic breast cancer upon completing active treatment for primary breast cancer can increase sense of control and psychological well-being. Our participants believed in the utility of the Infographics equipping them with knowledgeable tools that can empower them in the face of fears and anxieties regarding recurrence and progression of disease. Individual differences in the perceived impact of the Infographics on fears of recurrence and progression of disease were modulated by emotion regulation strategies such as expressive suppression and anxiety vulnerability. Tailored approaches can increase the effectiveness of educational programmes on signs and symptoms of metastatic breast cancer.

## Data availability statement

The raw data supporting the conclusions of this article will be made available by the authors, without undue reservation.

## Ethics statement

The studies involving humans were approved by Ethics Committee, School of Psychology and Clinical Language Sciences at the University of Reading (Reference number: 2023 – 021 – ND). The studies were conducted in accordance with the local legislation and institutional requirements. The participants provided their written informed consent to participate in this study.

## Author contributions

ND: Conceptualization, Data curation, Formal analysis, Funding acquisition, Investigation, Methodology, Project administration, Resources, Software, Supervision, Validation, Visualization, Writing – original draft, Writing – review & editing. JT: Conceptualization, Investigation, Methodology, Resources, Supervision, Validation, Visualization, Writing – review & editing. BC: Conceptualization, Data curation, Formal analysis, Investigation, Methodology, Project administration, Resources, Software, Validation, Visualization, Writing – review & editing.

## Footnotes

^1^ https://www.abcdiagnosis.co.uk/resources/infographics/

^2^ https://metupuk.org.uk/

^3^ www.gorilla.sc
