# Supplementary material for: Infographics on signs and symptoms of metastatic (secondary) breast cancer can empower women with a breast cancer diagnosis
Source: Front Psychol. 2024 Jul 12;15:1403114. doi: 10.3389/fpsyg.2024.1403114 (PMC11274755; doi:10.3389/fpsyg.2024.1403114)
Supplement: Supplementary file 1 [file Table_1.DOCX]

1. In your opinion, do you think that you were well informed about the signs of recurrence, secondary breast cancer, or progression by your oncology team?

| **0** | [Not at all](javascript:;) [A little](javascript:;) [Somewhat](javascript:;) [A lot](javascript:;) [A great deal](javascript:;) | **4** |
| --- | --- | --- |

1. In your opinion, did you want to know about the signs and symptoms of secondary breast cancer, recurrence or progression?

 Yes
 No
 Other (please specify)

1. In your opinion, do you feel like you were given the opportunity to discuss any concerns or worries you had about the signs and symptoms of secondary breast cancer, recurrence or progression with your oncology team following the completion of your active treatment for primary breast cancer?

| **0** | [Not at all](javascript:;) [A little](javascript:;) [Somewhat](javascript:;) [A lot](javascript:;) [A great deal-](javascript:;) | **4** |
| --- | --- | --- |

1. If you have had concerns, do you feel like your oncology team has answered your concerns or worries about the signs and symptoms of secondary breast cancer, recurrence or progression to your satisfaction?

| **0** | [Not at all](javascript:;) [A little](javascript:;) [Somewhat](javascript:;) [A lot](javascript:;) [A great deal](javascript:;) | **4** |
| --- | --- | --- |

1. In your opinion, were you satisfied with the level of information you were provided with by your oncologist at the end of your primary breast cancer treatment regarding the signs and symptoms of secondary breast cancer?

 Yes
 No
 Other (please specify)

**The next section of questions you will be asked will be about the infographics.**

1. In your opinion, do you think this infographic should be given to GPs to help promote their awareness, understanding, knowledge and learning of the signs and symptoms of secondary breast cancer?

| **0** | [Strongly disagree](javascript:;) [Disagree](javascript:;) [Undecided](javascript:;) [Agree](javascript:;) [Strongly agree](javascript:;) | **4** |
| --- | --- | --- |

1. Would you have liked to have received a copy of this infographic from your oncology team as part of your treatment summary?

| **0** | [Strongly disagree](javascript:;) [Disagree](javascript:;) [Undecided](javascript:;) [Agree](javascript:;) [Strongly agree](javascript:;) | **4** |
| --- | --- | --- |

1. In your opinion, do you think this infographic should be discussed by your oncologist so they can elude you to the different signs and symptoms when they provide you with a copy of this information:

| **0** | [Strongly disagree](javascript:;) [Disagree](javascript:;) [Undecided](javascript:;) [Agree](javascript:;) [Strongly agree](javascript:;) | **4** |
| --- | --- | --- |

1. In your opinion, do you think the information on the infographics is more helpful than the information you have received from your oncologist or oncology team?

| **0** | [Strongly disagree](javascript:;) [Disagree](javascript:;) [Undecided](javascript:;) [Agree](javascript:;) [Strongly agree](javascript:;) | **4** |
| --- | --- | --- |

1. In your opinion, do you think receiving a copy of this infographic would influence how likely you are to seek medical attention?

| **0** | [Strongly disagree](javascript:;) [Disagree](javascript:;) [Undecided](javascript:;) [Agree](javascript:;) [Strongly agree](javascript:;) | **4** |
| --- | --- | --- |

1. Do you think this infographic would influence how often you think about the possibility of cancer recurrence, secondary breast cancer or cancer progression?

| **0** | [Strongly disagree](javascript:;) [Disagree](javascript:;) [Undecided](javascript:;) [Agree](javascript:;) [Strongly agree](javascript:;) | **4** |
| --- | --- | --- |

1. Do you think receiving this infographic would make you feel less fearful about the possibility of secondary breast cancer or cancer progression?

| **0** | [Not at all](javascript:;) [A little](javascript:;) [Somewhat](javascript:;) [A lot](javascript:;) [A great deal](javascript:;) | **4** |
| --- | --- | --- |

1. Do you think receiving this infographic would make you worry less about secondary breast cancer or cancer progression?

| **0** | [Not at all less](javascript:;) [A little less](javascript:;) [Somewhat less](javascript:;) [A lot less](javascript:;) [Much less](javascript:;) | **4** |
| --- | --- | --- |

1. Do you think this infographic would make you feel more in control of your health?

| **0** | [Not at all](javascript:;) [A little](javascript:;) [Somewhat](javascript:;) [A lot](javascript:;) [A great deal](javascript:;) | **4** |
| --- | --- | --- |

1. In your opinion, do you think the information presented on the infographic would make you feel more empowered?

| **0** | Not at all [A little](javascript:;) [Somewhat](javascript:;) [A lot](javascript:;) [A great deal](javascript:;) | **4** |
| --- | --- | --- |

1. Do you think this infographic would help you cope with the uncertainty surrounding the risk of secondary breast cancer, cancer recurrence or progression?

| **0** | [Not at all](javascript:;) [A little](javascript:;) [Somewhat](javascript:;) [A lot](javascript:;) [A great deal](javascript:;) | **4** |
| --- | --- | --- |

1. In your opinion, do you think discussing this infographic with your oncologist would make you feel more knowledgeable about secondary breast cancer?

| **0** | [Not at all](javascript:;) [A little](javascript:;) [Somewhat](javascript:;) [A lot](javascript:;) [A great deal](javascript:;) | **4** |
| --- | --- | --- |

1. In your opinion, do you think knowing these signs will make you feel more equipped (prepared) to make decisions about your health?

| **0** | [Not at all](javascript:;) [A little](javascript:;) [Somewhat](javascript:;) [A lot](javascript:;) [A great deal](javascript:;) | **4** |
| --- | --- | --- |

Top of Form

1. What do you find positive about the design of the infographic (please list up to three points)
2. In your opinion, what do you think could be changed with the design or information included on the infographic (please list up to three points)
3. In your opinion, do you think any further information should be included in the infographic (please specify what information and why this would be helpful) (please list up to three points)
4. It is common to use terminology like “red flags” to point out potential indicators of disease. In your opinion, what do you think about the terminology “red flags”?

| **0** | [Not at all bothered](javascript:;) [A little bothered](javascript:;) [Somewhat bothered.](javascript:;) [A lot bothered](javascript:;) [A great deal bothered](javascript:;) | **4** |
| --- | --- | --- |

Bottom of Form
